# Controlled Growth of BiSI Nanorod-Based Films through a Two-Step Solution Process for Solar Cell Applications

**DOI:** 10.3390/nano9121650

**Published:** 2019-11-20

**Authors:** Yong Chan Choi, Eunjeong Hwang

**Affiliations:** Division of Energy Technology, DGIST, Daegu 42988, Korea; ejhwnag@dgist.ac.kr

**Keywords:** bismuth chalcohalides, BiSI, Bi_2_S_3_, solution process, thiol-amine, solar cells

## Abstract

Pb-based hybrid perovskite solar cells, despite their advantages, face challenges in commercialization. In recent years, Bi-based chalcohalides are being considered as potential alternative candidates, however, their current device efficiency remains unsatisfactory. Herein, a two-step solution method is developed and applied to the fabrication of BiSI films. The method consists of the formation of Bi_2_S_3_ (step I) and its conversion to BiSI (step II). The Bi_2_S_3_ was fabricated by a thiol-amine solution process and the BiSI conversion was achieved by chemical reaction between the as-formed Bi_2_S_3_ and BiI_3_. It was found that the formation of BiSI was highly dependent on the Bi:S molar ratio of the Bi_2_O_3_-thiourea solution and the number of times of step I. The as-fabricated BiSI film had an optical band gap of 1.61 eV and exhibited nanorod morphology. In addition, the electronic structure is explored and discussed for solar cells applications.

## 1. Introduction

Pb-based hybrid perovskites (Pb perovskites) solar cells have several advantages, such as remarkable device efficiency, low-cost fabrication, and unique optoelectronic properties [1,2,3]. Thus, these are now considered to be the most promising substitute for Si, widely used in the solar market. However, several challenges, such as scale-up and reproducibility issues in performance and the manufacturing process, as well as toxicity, must be solved to ensure success in the commercial market. Therefore, considerable efforts are being made to address these issues, or to develop tandem solar cells combined with other solar cells [1]. Researchers are also constantly looking for alternatives to Pb perovskites [4].

Recently, Bi-based chalcohalides, such as BiSI and BiSeI, are being considered as good alternatives because of their suitability as solar absorbers and stability [4,5,6,7,8,9,10]. In addition, these materials have a lower material cost compared to Pb perovskites (Supplementary Table S1) and low toxicity, due to non-toxic Bi content [5]. In particular, the solar cells based on them are expected to exhibit a high device efficiency because of the ns^2^ electronic configuration of Bi^3+^ (like Pb^2+^ of Pb perovskites), enabling defect-tolerant features [4,5,6]. Despite their great potential, little work has been carried out on solar cells, and their best reported device efficiency of 1.32% is unsatisfactory [8,9]. Therefore, further work is required to prove their potential as a photovoltaic material. However, fabrication methods suitable for solar cells are still lacking and have not been optimized to achieve the best performance.

BiSI and BiSeI have been fabricated using several methods, including a spray solution method [8], conversion reaction from BiOI particles under H_2_(S,Se) gas [10], and a single source precursor solution method [9]. These methods are simple, versatile, and cost-effective because they are based on a solution process and performed at a low temperature below 300 °C. However, these techniques have not been fully validated as suitable methods for solar cells. Therefore, it is still necessary to develop an approach that can control the key variables, such as the crystal structure, morphology, and optoelectronic properties. We recently developed a simple solution method for typical Sb chalcohalides, SbSI, via a two-step process [11]: (i) formation of Sb_2_S_3_ and (ii) its conversion to SbSI based on the simple chemical reaction of Sb_2_S_3_ + SbI_3_ → 3SbSI. With this method, the structure and morphology of SbSI were successfully controlled by tuning experimental parameters at each step. Given that SbSI is isostructural to BiSI [12], this two-step method can be easily applied to the BiSI fabrication by the following chemical reaction: Bi_2_S_3_ + BiI_3_ → 3BiSI. To this end, we chose the thiol-amine solution method for the Bi_2_S_3_ fabrication in step I, which turned out to be a very effective approach of preparing various chalcogenides [13,14].

In this work, we introduce the fabrication of BiSI films via our two-step solution process for solar cell applications. The Bi_2_S_3_ is first fabricated using the Bi_2_O_3_-thiourea (Bi_2_O_3_-TU) thiol-amine solution (step I), where Bi_2_O_3_ and TU are dissolved in 2-mercaptoethanol/ethanolamine (1/4 *v*/*v*) mixture. Then, it is converted to BiSI in the chemical reaction between as-formed Bi_2_S_3_ and BiI_3_ (step II). The structures, absorption, and morphology are controlled by tuning the Bi:S molar ratio of the Bi_2_O_3_-TU solution and the number of repetitions of step I. The morphology of as-fabricated BiSI film exhibits nanorods rather than a compact film. In addition, the electronic structure of BiSI is investigated and discussed for solar cell applications.

## 2. Materials and Methods

### 2.1. Chemicals and Materials

Bi_2_O_3_ (99.999%), BiI_3_ (99.999%), 2-mercaptoethanol (98%), and ethanolamine (98%) were purchased from Alfa Aesar. Thiourea (TU; >99.0%), N-methyl-2-pyrrolidinone (NMP; anhydrous, 99.5%) and 1,2-dichlorobenzene (DCB; anhydrous 99%) were purchased from Sigma-Aldrich. All chemicals were used as received without further purification. A pre-patterned F-doped SnO_2_ (FTO) substrate (Pilkington, TEC-8, 8 Ω/sq, 25 mm × 25 mm) with a FTO-etched surface area of 5 mm × 10 mm was purchased from AMG (Korea). A stable 0.15-M TiO_2_ solution was purchased from ShareChem (Korea).

### 2.2. Synthesis of Bi_2_O_3_-TU and BiI_3_ Solutions

To synthesize the Bi_2_O_3_-TU solution based on Bi_2_O_3_ and TU, Bi_2_O_3_ (0.3 mmol) was first dissolved in 1 mL of the thiol-amine mixture solvent (1:4 *v*/*v* of 2-mercaptoethanol:ethanolamine) and stirred for 1 day until a stable solution was obtained. Then, the desired amount of Bi_2_O_3_ solution was added to the TU-containing vial and stirred for 1 day to obtain the Bi_2_O_3_-TU solution with the desired Bi:S molar ratio. For the synthesis of the BiI_3_ solution, 1 mmol of BiI_3_ was immediately dissolved in 1 mL of NMP by vigorous stirring. These synthetic procedures were performed in a glove box.

### 2.3. Deposition of BiSI Thin Films

The 0.15M-TiO_2_ solution was spin coated on the FTO substrate at 5000 rpm for 60 s and dried at 200 °C for 2 min. Before deposition, the FTO substrate was cleaned with ultrasonic cleaner using acetone/ethanol and UV/O_3_ cleaner [15] and the TiO_2_ solution was filtered using a 0.45 µm polyvinylidene fluoride filter. The spin coating and drying steps were repeated 3 times. Then the as-deposited substrate was annealed at 500 °C in air for 30 min, to form the anatase TiO_2_ blocking layer (TiO_2_-BL) on the FTO substrate. After the TiO_2_-BL/FTO was cooled to room temperature, it was treated with a UV/O_3_ cleaner for 20 min and immediately transferred to the glove box for the BiSI deposition.

BiSI thin films were deposited via two-step solution process in the glove box. In step I, as-prepared Bi_2_O_3_-TU solution was spin coated on TiO_2_-BL/FTO at 5000 rpm for 70 s and dried at 200 °C for 5 min to form Bi_2_S_3_/TiO_2_-BL/FTO. In step II, BiI_3_ solution was dripped on Bi_2_S_3_/TiO_2_-BL/FTO and spin coated with the same condition used in step I. Then the BiI_3_-deposited Bi_2_S_3_/TiO_2_-BL/FTO was annealed at 200 °C for 30 min for the conversion of Bi_2_S_3_ to BiSI. Finally, after the sample was cooled to room temperature, a NMP solvent was dripped and spin coated to remove any residual BiI_3_, which has not contributed to the BiSI formation, from the surface. Note that this final step is crucial for obtaining pure BiSI structures because a residual BiI_3_ causes the formation of undesirable phases (Supplementary Figure S1).

### 2.4. Characterization

The structure was measured with a multi-purpose X-ray diffractometer (Empyrean, Malvern Panalytical Ltd., Malvern, UK). Its structural phase was identified using software (X’Pert HighScore Plus) and indicated with a reference code number in the text. The absorption, morphology, and electronic structure were investigated using a UV-vis spectrophotometer (UV-2600, Shimadzu Corp, Kyoto, Japan), a field emission scanning electron microscope (S-4800, Hitachi Ltd., Tokyo, Japan), and an X-ray photoelectron spectrometer (ESCALAB 250Xi, Thermo Fisher Scientific Inc., MA, USA), respectively.

## 3. Results and Discussion

**Figure 1:**
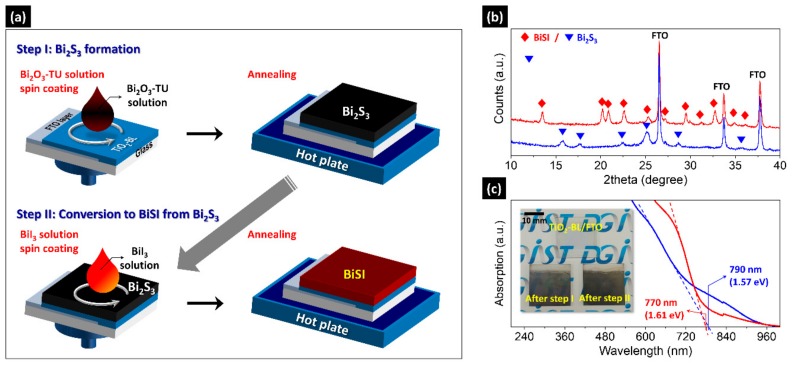
(**a**) Schematic diagram of the BiSI fabrication via the two-step solution process; (**b**) X-ray diffraction (XRD) patterns; (**c**) absorption spectra of the samples prepared after step I (blue line) and step II (red line). Inset in (**c**): photograph of TiO_2_-BL/FTO and the two samples shown in (**b**,**c**). The samples were fabricated with the Bi_2_O_3_-TU solution of Bi:S = 1:3 molar ratio on TiO_2_-BL/FTO substrates.

Figure 1a shows the schematic diagram of the BiSI fabrication process. The samples fabricated after step I and II exhibit orthorhombic Bi_2_S_3_ (reference code: 98-061-7028) and orthorhombic BiSI (reference code: 98-002-3631) phases, respectively, as shown in Figure 1b. The optical band gaps *E_G_* of Bi_2_S_3_ and BiSI are measured as 1.57 eV and 1.61 eV, respectively. These are close to the reported values of 1.3–1.7 [16,17] and 1.5–1.8 eV [5,6,7,8,9,18,19], respectively. They have similar absorption edges around 1.6 eV (Figure 1c), thereby showing a similar sample color, as shown in the inset image. These results indicate that BiSI film was successfully obtained from the Bi_2_S_3_ film at a low temperature of 200 °C via our two-step solution process.

**Figure 2:**
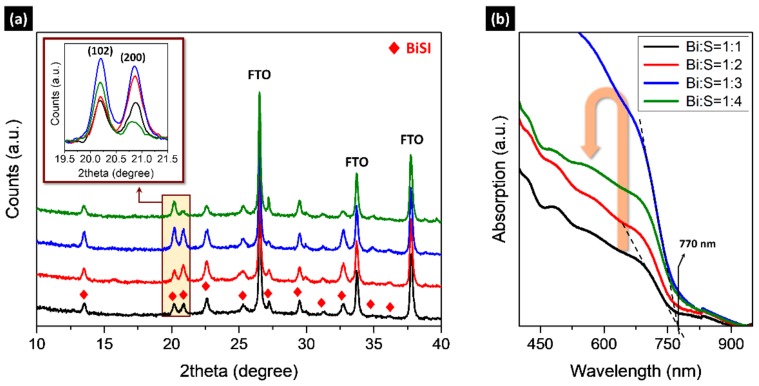
(**a**) XRD patterns and (**b**) absorption spectra of the BiSI thin films fabricated Bi_2_O_3_-TU solution of various Bi:S molar ratios. All samples were fabricated on TiO_2_-BL/FTO.

We found that the Bi:S molar ratio of the Bi_2_O_3_-TU solution, used in step I, strongly affects the BiSI formation. We controlled the molar ratio of the solution based on the stoichiometric molar fraction of Bi/S = 1/1.5 for Bi_2_S_3_. Figure 2a shows XRD patterns of the BiSI thin films fabricated with the same conditions as the sample shown in Figure 1 but using Bi_2_O_3_-TU solution of different Bi:S molar ratios. All samples exhibit a pure orthorhombic BiSI phase but show a different intensity for each peak. To clearly display the difference in peak intensity of each pattern, we highlighted two peaks corresponding to (102) and (200) planes, shown in the inset image. The intensity of the two peaks gradually increased as the amount of S increased in Bi:S, from 1:1 to 1:3, and then decreased at Bi:S = 1:4. The absorption spectra (Figure 2b) exhibited similar trends, where the absorption edges were the same regardless of the ratio and consistent with the value of Figure 1c. These results indicate that the Bi:S ratio plays a key role in BiSI formation.

The different BiSI formation by the Bi:S molar ratio may be explained in terms of the contribution of excess TU to BiSI formation. The TU as a sulfur source should be sufficiently supplied at each step to compensate for the loss of volatile TU at our annealing temperature of 200 °C. In addition, an excess TU may contribute to stabilizing the Bi-TU complex in the solution [11,20], enabling a stable supply of S. Thus, the BiSI formation is more enhanced as the more TU is supplied as shown in Figure 2. However, at a highly excess TU condition, organic residues derived from residual TU may interfere with BiSI formation. As a result, the BiSI with maximum intensity can be obtained at the specific ratio of Bi:S = 1:3. Interestingly, the optimum ratio of Bi:S (1:3) is same as that of Sb:S used for SbSI [11], although the solution method and the material are quite different. Besides, the TU plays a similar role in forming the crystalline phase in both materials. Therefore, we can expect that the optimum ratio condition may be useful in the formation of other Sb/Bi chalcohalides, such as SbSeI and BiSeI.

**Figure 3:**
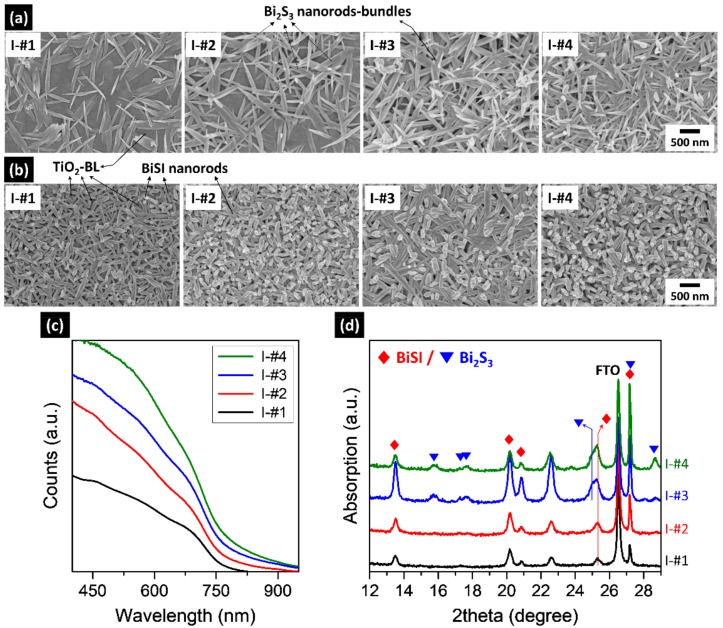
Effects of the number of repetitions of step I: Field emission scanning electron microscopy (FESEM) surface images of the samples prepared after (**a**) step I; (**b**) step II; (**c**) absorption spectra and (**d**) XRD patterns of the samples prepared after step II. In figures, I-#number indicates the number of repetitions in step I. The samples were fabricated on TiO_2_-BL/FTO using the Bi_2_O_3_-TU solution (Bi:S = 1:3 ratio).

The BiSI formation could also be controlled by adjusting the repetition of step I. The samples were fabricated under the same conditions as the sample of Figure 1, except for the number of repetitions of step I. As shown in Figure 3a,b, and Supplementary Figure S2, Bi_2_S_3_ bundles consisting of nanorods with 10–30 nm diameters, were formed after step I and converted to BiSI nanorods in step II. The diameter (60–100 nm) of BiSI nanorods were larger than that of the Bi_2_S_3_ nanorods, suggesting that the Bi_2_S_3_ aggregates to form BiSI nanorods during step II. These results indicate that the BiSI film consists of nanorods rather than a continuous film. As the number of repetitions increased, the number of Bi_2_S_3_ bundles increased, as shown in Figure 3a. As a result, more BiSI nanorods were formed in step II. Simultaneously, the thickness of the BiSI nanorod film was increased from 210 to 657 nm (Supplementary Table S2). Thus, a large number of nanorods covered the entire surface after three repetitions (Figure 3b and Supplementary Figure S3). This increased number of nanorods was supported by enhanced absorption with increasing repetitions (Figure 3c). To further confirm this, structures were investigated with XRD. The results are shown in Figure 3d. Up to three repetitions (I-#3), the peak intensity of BiSI phase increased, but decreased when step I was repeated four times (I-#4). Simultaneously, the Bi_2_S_3_ phase appeared at I-#3 and its intensity was further enhanced at I-#4. This result indicates that some of Bi_2_S_3_ remained unconverted to BiSI in the two samples for I-#3 and I-#4. Thus, improved absorption observed at I-#3 and I-#4 can be explained by the contribution of BiSI and Bi_2_S_3_ rather than just BiSI, because both have similar absorption edges as shown in Figure 1c. Therefore, pure-phase BiSI with maximum intensity could be obtained when step I was repeated twice.

**Figure 4:**
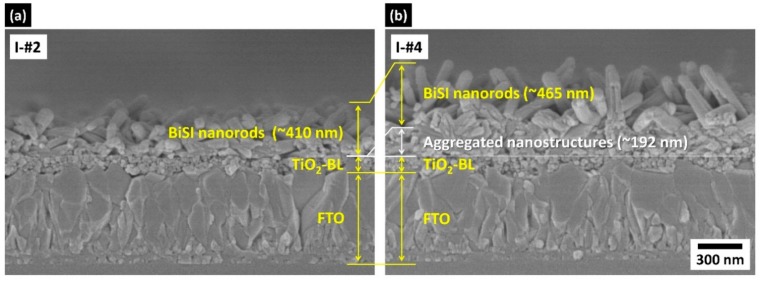
Cross-sectional FESEM images of (**a**) I-#2 and (**b**) I-#4 samples prepared after step II.

Note that the repetition of step II did not cause further conversion of Bi_2_S_3_ to BiSI, formed in the I-#3 and I-#4 samples, but resulted in BiOI formation (Supplementary Figure S4). To figure out the reason why BiSI was no longer converted from the Bi_2_S_3_ in I-#3 and I-#4, we examined their morphology and compared it with those obtained from the I-#1 and I-#2 samples. As show in Figure 3a and Supplementary Figure S2, the Bi_2_S_3_ bundles formed after step I, in I-#1 and I-#2, did not cover the entire surface of underlying TiO_2_-BL, revealing a portion of the TiO_2_-BL surface. The converted samples show the nanorods randomly grown on the TiO_2_-BL/FTO substrate (Figure 4a). As a result, the nanorods were grown from 210 to 410 nm as step I was repeated (Table S2). In contrast, the Bi_2_S_3_ bundles were intricately intertwined so that the underlying TiO_2_-BL surface was not revealed in I-#3 and I-#4 (Figure 3a). The samples obtained from such intertwined bundles exhibit a two-layered structure, with nanorods on the upper layer and aggregated nanostructures on the lower layer (Figure 4b). The thickness of each layer is very similar in I-#3 and I-#4 samples, as revealed in Table S2, suggesting that the multiple coating did not affect the BiSI growth. By comparing morphologies and structures, it can be inferred that the nanorods in the upper layer and nanostructures in the lower layer mainly consisted of BiSI and Bi_2_S_3_, respectively. This was confirmed by the grazing incident XRD (GIXRD) measurement (Supplementary Figure S5). These results indicate that the intertwined Bi_2_S_3_ bundles prevented the BiI_3_ solution from reaching the bottom of the Bi_2_S_3_ layer, in the I-#3 and I-#4 samples. Thus, the Bi_2_S_3_ at the bottom could react with BiI_3_, leaving unconverted Bi_2_S_3_ at the bottom. As a result, the BiSI nanorods were formed in the upper layer, in which the BiI_3_ solution can actively react, whereas the nanostructures mainly composed of Bi_2_S_3_ are formed in the lower layer.

**Figure 5:**
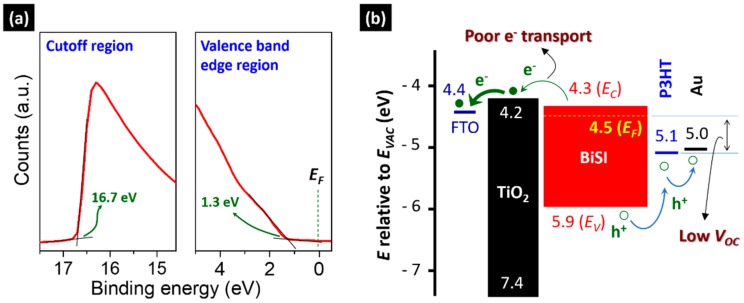
(**a**) UPS spectra of the cut-off and valence band edge region for the BiSI nanorods film, and (**b**) the derived energy-level band diagram. For comparison, a conducting oxide (FTO), ETL (TiO_2_), HTM (P3HT), and electrode (Au) were drawn based on a previous study [11].

To investigate the electronic structure of the BiSI thin film, we measured ultraviolet photoemission spectroscopy (UPS) spectra, as shown in Figure 5a. From the two spectra of the cutoff and valence band edge regions, we could obtain the cutoff energy *E_cutoff_* and difference Δ = *E_V_* - *E_F_*; where *E_V_* is the valence band maximum and *E_F_* is Fermi level. These were 16.7 and 1.3 eV, respectively. Correspondingly, we could calculate *E_F_*, *E_V_*, and conduction band minimum (CBM, *E_C_*), which were 4.5, 5.9, and 4.3 eV, respectively, based on the relation of *E_F_* = hν – (*E_cutoff_ − E_VAC_)* (*E_VAC_*: vacuum level energy) and *E_G_*. From these results, it was revealed that the BiSI behaves like a n-type semiconductor, consistent with a previous study [8], because *E_F_* is located close to *E_C_*. Based on this measurement, we drew an energy-level diagram for the as-fabricated BiSI nanorods film, as shown in Figure 5b. We also included other widely used layers to examine its applicability to solar cells. For a solar cell that constructed with FTO, TiO_2_, poly(3-hexylthiophene) (P3HT) as the conducting oxide, electron transporting layer (ETL), and hole transporting material (HTM), respectively, we expected very poor device performance. A preliminary result confirmed that this device did not work properly (Supplementary Figure S6 and Table S2). These are for the following two reasons: First, it is very difficult for electrons to transfer from the CBM of BiSI to the CBM of TiO_2_ because the CBM of BiSI is located below that of TiO_2_. Second, the highest occupied molecular orbitals (HOMO) level of P3HT is located at 5.1 eV; ~0.6 eV lower than *E_F_* of BiSI. Thus, the maximum open circuit voltage *V_OC_* is limited to 0.6 eV. Therefore, to achieve a high device efficiency, these two issues must be solved.

The first issue can be addressed by using an alternative ETL that has a CBM lower than that of BiSI. Material engineering via doping/alloying for shifting the CBM of BiSI could be another solution for solving this, while maintaining the TiO_2_ ETL. To address the second issue, a HTM with a deep HOMO level should be used, such as 3,3’-dimethoxybenzidine moieties (S9, 5.5 eV) [21], poly(9,9-di-n-octylfluorenyl-2,7-diyl) (F8, 5.8 eV) [5,9] and poly(9,9-dioctylfluorene-alt-benzothiadiazole) (F8BT, 5.9 eV) [22]. Very recently, Tiwari et al. addressed these issues by using SnO_2_ and F8 as the ETL and HTM, respectively, and achieved the best device efficiency (1.32%) in BiSI solar cells [9]. This efficiency is a significant improvement compared to 0.012% for the solar cell fabricated by Hahn et al [8]. Despite great progress in performance, the best efficiency is still very far below that of Pb perovskites solar cells. Therefore, several other factors, such as defects, crystalline orientation, device architecture, and material engineering, should also be considered to further enhance the performance [5,6].

## 4. Conclusions

BiSI films with *E_G_* = 1.61 eV were fabricated via our two-step solution process which consists of Bi_2_S_3_ formation and its conversion to BiSI at a low temperature of 200 °C. Through controlling experimental parameters, the best crystallinity of BiSI could be obtained with the molar ratio of Bi:S = 1:3 and two repetitions of step I. As-fabricated BiSI showed nanorods, 60–100 nm in diameter, grown randomly on TiO_2_-BL/FTO, rather than a continuous film. In addition, we found that the BiSI behaved like a n-type semiconductor with *E_F_* 4.5 eV, *E_V_* 5.9 eV, and *E_C_* 4.3 eV. Our work is expected to contribute to improving the performance of Bi-based chalcohalide solar cells and to provide some clues for developing low-cost and environment-friendly solar cells.

## Supplementary Materials

The following are available online at https://www.mdpi.com/2079-4991/9/12/1650/s1, Table S1: comparison of costs for fabrication of BiSI and CH_3_NH_3_PbI_3_, Figure S1: XRD pattern of bare sample and treated with NMP, Figure S2: high magnification FESEM surface images of the sample prepared after step I and step II, Figure S3: low magnification FESEM surface image of the BiSI nanorods (I-#4 sample) formed on TiO_2_-BL/FTO, Table S2: thickness of the BiSI films shown in Figure 3 and Figure 4, Figure S4: effect of step II repetitions on the sample (I-#3) shown in Figure 3, Figure S5: GIXRD patterns as a function of incident angle α and normal XRD pattern of the sample shown in Figure 4b, Figure S6. cross-sectional FESEM images of the solar cells based on (a) BiSI and (b) Pb perovskite, Table S2. Device parameters of two samples are shown in Figure S6.
