# Supplementary material for: Controlled Growth of BiSI Nanorod-Based Films through a Two-Step Solution Process for Solar Cell Applications
Source: Nanomaterials (Basel). 2019 Nov 20;9(12):1650. doi: 10.3390/nano9121650 (PMC6956077; doi:10.3390/nano9121650)
Supplement: Supplementary file 1 [file nanomaterials-09-01650-s001.pdf]

Supplementary Material *for*

Controlled Growth of BiSI Nanorod-Based Films Through a Two-Step Solution Process for Solar Cell Applications

Yong Chan Choi* and Eunjeong Hwang

Division of Energy Technology, DGIST, Daegu 42988, Republic of Korea

Component	Raw material	Price ($/g) [a]	Material cost ($/g) [b]
**BiSI**	BiI_3 99.998%	15.2	16.9 [b-1]
	Bi_2O_3 99.999%	4.6	
	TU >99%	0.4	
**$CH_3NH_3PbI_3$**	PbI_2 99.999%	22.2	49.0 [b-2]
	CH_3NH_3I	26.8	

Table S1. Comparison of costs for fabrication of BiSI and $CH_3NH_3PbI_3$. This was roughly calculated based on the material cost.

[a] Information on price was obtained from the homepage of Sigma-Aldrich.

[b] We calculated the material cost based the molar ratio for fabrication.

[b-1] In this work, 1 mol BiI_3, 0.3 mol Bi_2O_3, and 0.9 mol TU were used for fabrication of optimized BiSI thin film.

[b-2] Generally, 1 mol PbI_2 and 1 mol CH_3NH_3I were used for fabrication of $CH_3NH_3PbI_3$ thin film.

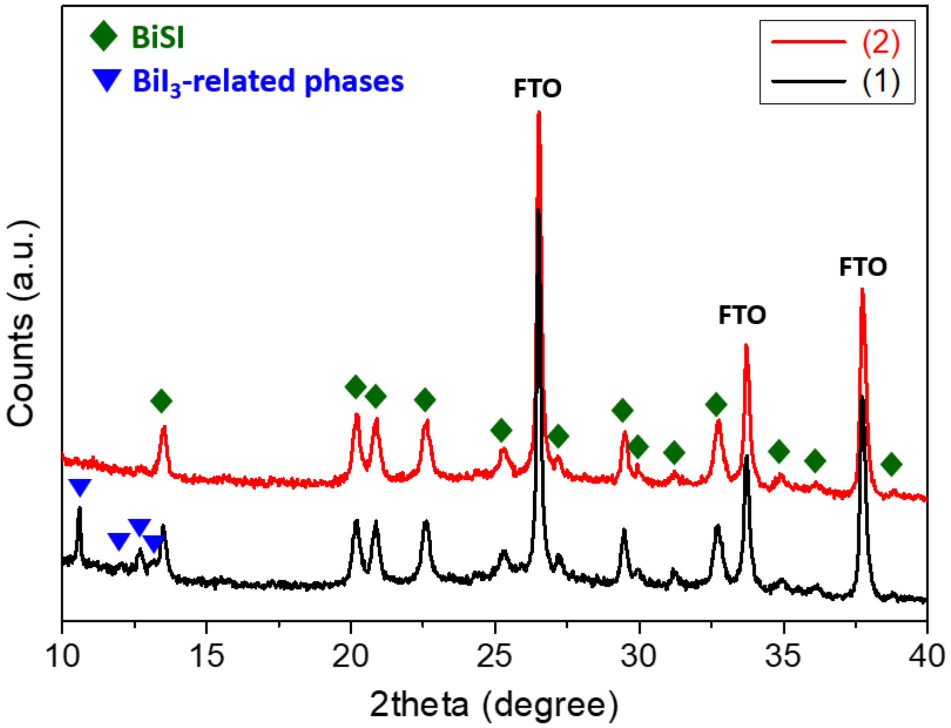

Figure S1. XRD pattern of bare sample (1) and treated with NMP (2). BiI_3-related phases, such as BiI_3 and $Bi_2I_4O_{13}$, are detected in addition to the BiSI phase from the bare sample.

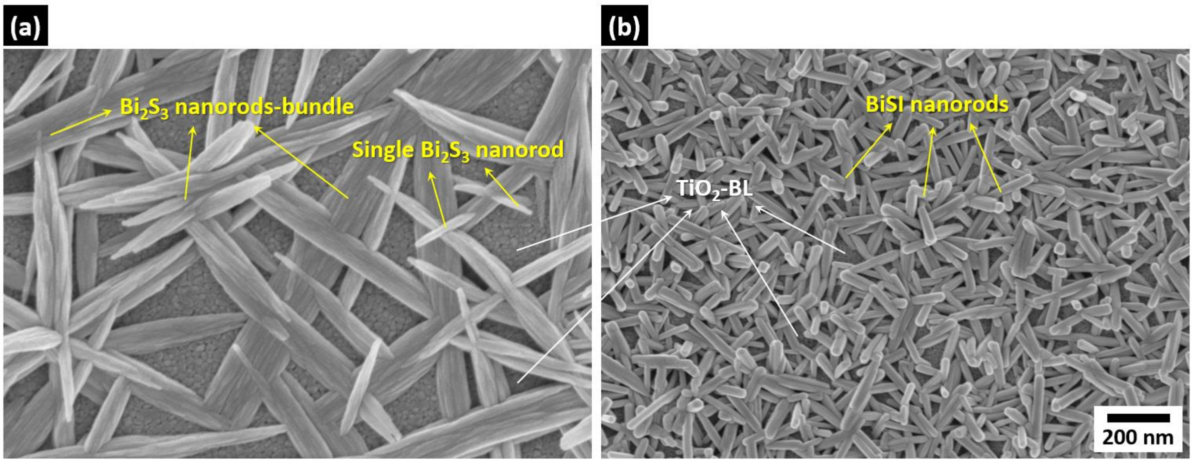

Figure S2. High magnification FESEM surface images of the sample prepared after (a) step I and (b) step II. The samples were fabricated on TiO_2-BL/FTO with the Bi_2O_3-TU solution (Bi:S=1:3).

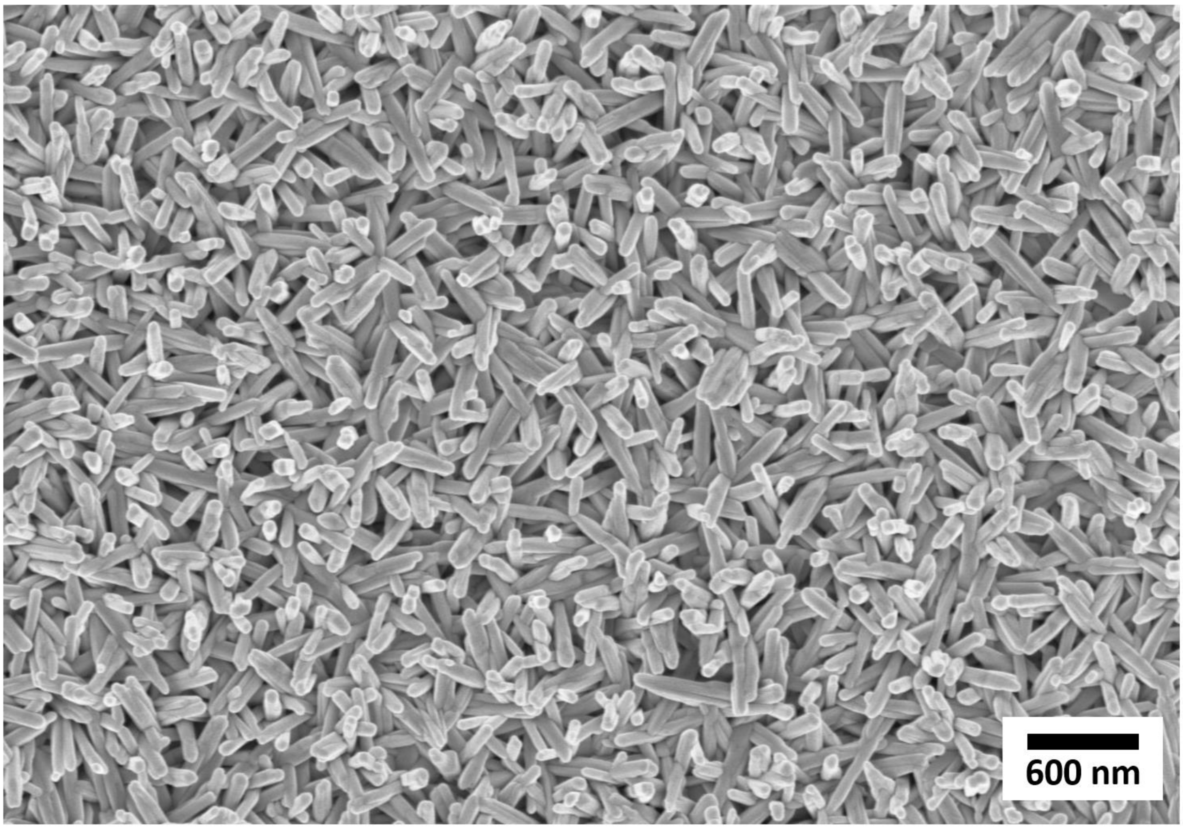

Figure S3. Low magnification FESEM surface image of the BiSI nanorods (I-#4 sample) formed on TiO_2-BL/FTO.

Thickness (nm)*	Sample			
	**I-#1**	**I-#2**	**I-#3**	**I-#4**
Nanorods layer	210	410	462	465
Aggregated nanostructures layer	-	-	190	192
Total layer	210	410	652	657

* The thickness was approximately measured based on the cross-sectional FESEM images.

Table 2. Thickness of the BiSI films shown in Figures 3 and 4.

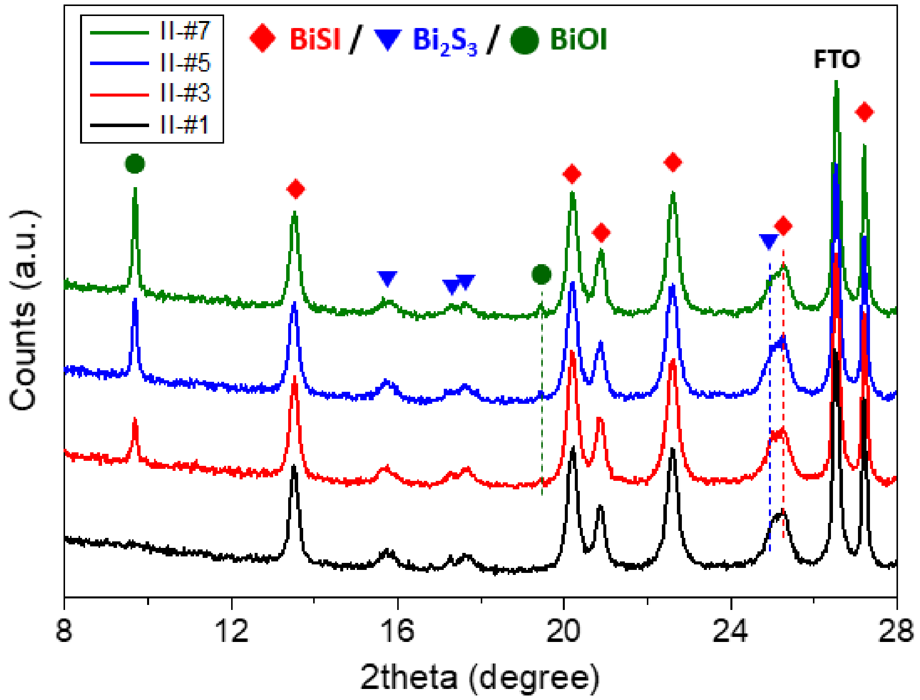

Figure S4. Effect of step II repetitions on the sample (I-#3) shown in Figure 3. In the pattern, II-#number indicates the number of repetitions in step II. No BiOI phase was observed in the pattern of the original sample (I-#3). However, as the number of step II repetitions increases, BiOI phase is increased while Bi_2S_3 and BiSI phases remain constant. The BiOI may be formed by an oxidation of residual BiI_3 on the surface.

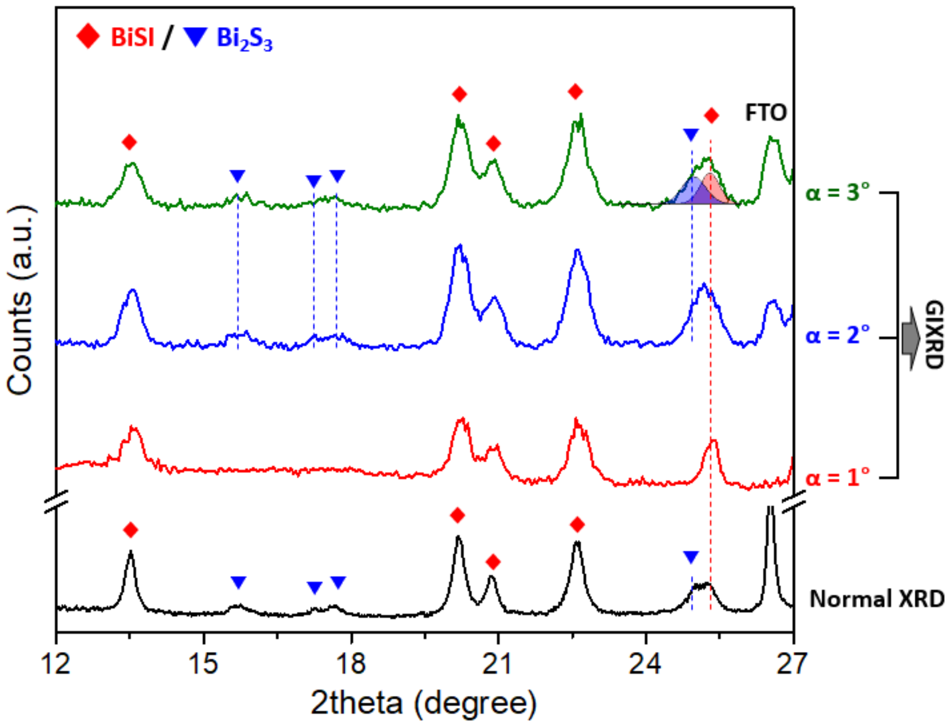

Figure S5. GIXRD patterns as a function of incident angle α and normal XRD pattern of the sample shown in Figure 4b. The structural change, with thickness, can be detected because the GIXRD measurement is performed at a fixed angle of incidence while the detector moves over the 2theta range of interest. In other words, the structural information near the surface can be obtained from the pattern measured at a low incident angle, and structural information at a deeper level can be measured as the angle increases. Only the BiSI phase is detected at a low angle of 1°, whereas both BiSI and Bi_2S_3 phases are observed at relatively higher angles of 2 and 3°. Therefore, it can be inferred that the upper region of the film mainly consists of BiSI.

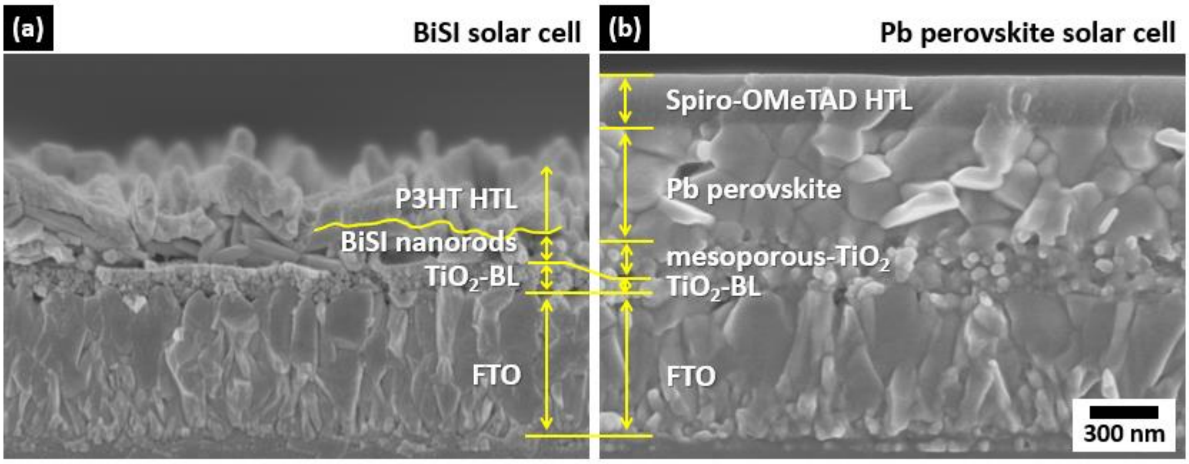

Figure S6. Cross-sectional FESEM images of the solar cells based on (a) BiSI and (b) Pb hybrid perovskite. For fabrication of BiSI solar cell, the BiSI was fabricated on TiO_2-BL/FTO with the same condition as the sample I-#2 of Figure 3. Then P3HT and Au were sequentially deposited by spin coating and thermal evaporation, respectively [11]. The Pb perovskite solar cell was fabricated according to our previous work [*APL Mater.* **2017**, *5*, 026101].

Solar cell	J_{SC} (mA cm^{-2})	V_{OC} (mV)	FF (%)	PCE (%)
BiSI	0.1	0.4	11.1	4.4×10^{-6}
Pb perovskite	21.0	1082.5	70.0	16.0

Table S3. Device parameters of two samples shown in Figure S6.

Unlike Pb perovskite solar cell, P3HT hole transporting layer (HTL) was not uniformly deposited on BiSI nanorod film in the BiSI solar cell due to the nanorod morphology. This may cause an undesirable direct contact between TiO_2-BL and P3HT HTL. In addition, there are still two problems, poor electron transfer and a higher HOMO level, as described in the last two paragraphs of the 'Results and discussion' section. Thus, the device efficiency of the BiSI solar cell is very poor compared to the Pb perovskite solar cell (Table S3).
